# Targeting *SLC7A11*/xCT improves radiofrequency ablation efficacy of HCC by dendritic cells mediated anti‐tumor immune response

**DOI:** 10.1002/imt2.248

**Published:** 2024-11-20

**Authors:** Yuzhao Jin, Songhua Cai, Yang Zhou, Dandan Guo, Yuzhen Zeng, Wangting Xu, Yiting Sun, Yueli Shi, Zhiyong Xu, Zaoqu Liu, Peng Luo, Zhao Huang, Bufu Tang

**Affiliations:** ^1^ Postgraduate Training Base Wenzhou Medical University Wenzhou China; ^2^ Department of Thoracic Surgery National Cancer Center/National Clinical Research Center for Cancer/Cancer Hospital & Shenzhen Hospital, Chinese Academy of Medical Sciences and Peking Union Medical College Shenzhen China; ^3^ Department of Gynecologic Oncology Zhongshan Hospital, Fudan University Shanghai China; ^4^ Department of Oncology First Affiliated Hospital, Dalian Medical University Dalian China; ^5^ Department of Radiation Oncology Zhongshan Hospital Affiliated to Fudan University Shanghai China; ^6^ Department of Respiratory Medicine Sir Run Run Shaw Hospital, Zhejiang University Hangzhou China; ^7^ Department of Clinical Medicine China Medical University Shenyang China; ^8^ Department of Respiratory and Critical Medicine Center for Oncology Medicine, The Fourth Affiliated Hospital of School of Medicine, International Institutes of Medicine, Zhejiang University Yiwu City China; ^9^ Institute of Basic Medical Sciences Chinese Academy of Medical Sciences and Peking Union Medical College Beijing China; ^10^ Department of Oncology Zhujiang Hospital, Southern Medical University Guangzhou Guangdong China; ^11^ Hepatic Surgery Center Tongji Hospital, Tongji Medical College, Huazhong University of Science and Technology Wuhan China; ^12^ Hubei Key Laboratory of Hepato‐Pancreatic‐Biliary Diseases Tongji Hospital, Tongji Medical College, Huazhong University of Science and Technology Wuhan China

## Abstract

After RFA treatment in patients with liver cancer, the expression of *SLC7A11*/xCT and the proportion of DCs in the TME were significantly increased. *SLC7A11*/xCT is a poor prognostic marker for liver cancer and is mainly expressed in DCs in the TME. Targeting xCT in DCs combined with RFA significantly enhances anti‐tumor immunity, suppressing tumor growth and offering a promising strategy for improved therapeutic outcomes in liver cancer.

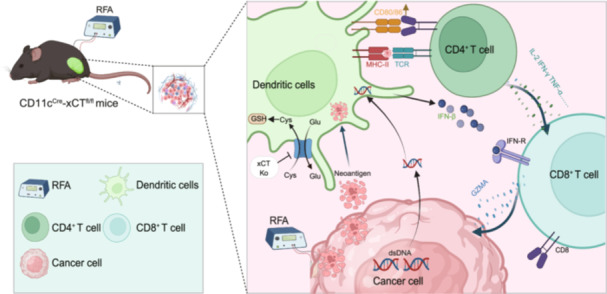

To the Editor,

Radiofrequency ablation (RFA) is widely used in clinical practice as a well‐established treatment for hepatocellular carcinoma (HCC) [1]. It can generate high temperatures within the tumor, inducing tumor cell necrosis, while also altering the tumor microenvironment (TME) to impact the local immune response [2]. However, as a standalone treatment, RFA often results in incomplete ablation of larger tumors, leading to a high risk of tumor recurrence [3]. Importantly, tumor fragments generated by RFA can trigger anti‐tumor immune responses, indicating that combining RFA with immunological therapies may improve outcomes in HCC patient [4].

Dendritic cells (DCs), as the most effective antigen‐presenting cells, serve as a crucial bridge between innate and adaptive immunity. In the TME, DCs can capture and process tumor antigens, presenting them to T cells and activating a specific immune response against the tumor [5, 6]. Studies have shown that the number of DCs markedly upregulated in the TME post‐ablation, especially near the ablation site, suggesting their potential role in RFA‐induced anti‐tumor immunity [7, 8]. Therefore, combining RFA with immunotherapies that enhance DCs' function may further amplify the anti‐tumor immune response and improve tumor regression.

The cellular redox balance is essential for anti‐tumor efficacy, and targeting oxidative stress can modulate immune cell functions [9]. The *SLC7A11*‐encoded xCT protein facilitates cysteine and glutamate transport, thereby regulating redox balance through intracellular glutathione synthesis. In addition, overexpression of SLC7A11 is associated with a poor prognosis in various cancers, suggesting its important role in tumor progression and drug resistance [10, 11]. However, research has predominantly concentrated on the antioxidant and anti‐ferroptotic roles of SLC7A11 in tumor cells, with its function in immune cells within the TME remaining elusive [12]. Here, we use comprehensive bioinformatics analyses and experimental validations to investigate SLC7A11's role in the liver cancer immune microenvironment, elucidate the underlying regulatory mechanisms, and offer insights on the RFA combination therapy model for HCC.

## RESULTS

### Upregulation of *SLC7A11*/xCT post‐ablation in DCs

**Figure 1:**
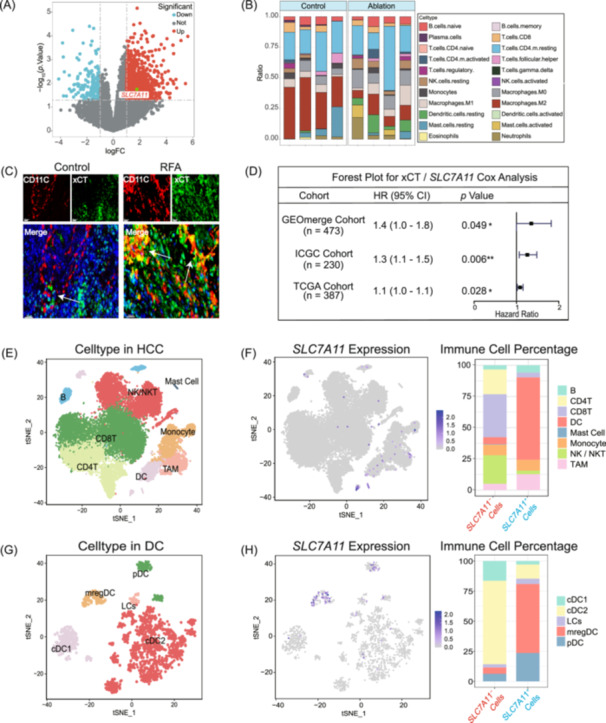
RFA upregulates xCT in dendritic cells (DCs) and *SLC7A11* is highly expressed in DCs. (A) Volcano plot showing upregulation of *SLC7A11* in liver cancer tissues post‐radiofrequency ablation (RFA). (B) Stacked bar chart illustrating increased DCs proportion after liver tumor ablation. (C) Immunofluorescence confirming upregulated xCT expression in DCs after RFA. (D) Univariate Cox analysis across multiple cohorts identifying *SLC7A11* as a poor prognostic marker in hepatocellular carcinoma (HCC). (E) tSNE map depicting the immune landscape of HCC. (F) tSNE and bar charts indicating high *SLC7A11* expression in DCs and the highest proportion of *SLC7A11* ^+^ cells within the DC population. (G) tSNE map showing DCs subtypes in HCC. (H) tSNE and bar charts demonstrating high *SLC7A11* expression in mregDC and the highest proportion of *SLC7A11* ^+^ cells among mregDC. [Correction added on 29 November 2024, after first online publication: Figure 1 has been replaced with the updated figure]

In our study, we initially compared the transcriptome sequencing data from liver cancer tissues pre‐ and post‐ablation. Our findings revealed a significant upregulation of xCT post‐ablation (Figure 1A). Analysis of immune cell infiltration demonstrated a notable increase in the proportion of DCs within the TME subsequent to ablation (Figure 1B and Figure S1A,B). Comparatively, at the basal protein expression level, xCT expression was more pronounced in DCs than in hepatic cancer cells or macrophages (Figure S1C). Subsequently, we extracted primary bone marrow‐derived dendritic cell (BMDCs) from mice and heat‐treated them. We observed a positive correlation between heat treatment temperature and the elevation of xCT protein expression (Figure S1D).

Based on the above findings, we speculated that RFA could increase the expression of xCT in DCs. Subsequently, we established a mouse model of subcutaneous liver cancer and administered RFA treatment. Western blot analysis confirmed an increase in xCT expression within the subcutaneous tumor tissues following RFA treatment (Figure S1E). Further immunofluorescence co‐localization staining evidenced that xCT upregulation post‐RFA was predominantly localized to DCs (Figure 1C and Figure S1F). Given the correlation between high *SLC7A11* levels and poor prognosis in HCC (Figure 1D and Figure S1G), it is necessary to further investigate the role of *SLC7A11*/xCT in DCs.

### Single‐cell transcriptome analysis reveals that *SLC7A11* plays an immunosuppressive role in DCs

To understand the role of *SLC7A11* in DCs, we extracted normal tissues and tumor tissues from liver cancer patients in the GSE140228 single‐cell transcriptome and analyzed them. The sample distribution and tissue distribution shown in Figure S2A,B indicate that the data integration and batch correction of the samples are effective. In the initial cell clustering, we divided all cells into 8 clusters: CD4T cells, CD8T cells, B cells, NK/NKT cells, DCs, TAMs, mast cells, and monocytes (Figure 1E). The specific markers based on the clustering are shown in Figure S2C.

We observed the expression of *SLC7A11* in the immune cell population and found that the overall expression was not high, but the proportion of DCs in the *SLC7A11* ^+^ cell composition was close to 70%, suggesting that *SLC7A11* may mainly play a role in TME through DCs (Figure 1F). Through standardized analysis process clustering and subgroup marker gene alignment, we identified five DC subgroups, namely, cDC1, cDC2, pDC, LCs, and mregDC (Figure 1G). The specific markers for clustering and the top five genes of each group are shown in Figure S2D,E, respectively. Among them, cDC1 highly expressed *CLEC9A*, which is closely related to cross‐presentation function; cDC2 highly expressed *CLEC10A*, which is closely related to T cell activation; pDC highly expressed *LILRA4*, which is related to antiviral infection; LCs specifically expressed *CD207*, which is related to skin antigen presentation; and mregDC highly expressed *CCR7* and *LAMP3*, which regulate the activity of T cells and play a role in immunosuppression [13] (Figure S2F). Although the proportion of DC in tumor tissue and normal tissue is not much different, mregDC seems to be expressed only in tumor tissue, suggesting its specificity in tumor immunity (Figure S3A,B). Interestingly, we found that *SLC7A11* is highly expressed primarily in mregDC, with lower expression in other types of DCs (Figure 1H). Additionally, its expression is upregulated in the later pseudotime, potentially reflecting the adaptive regulation of mregDC in the tumor microenvironment (Figure S3C).

To explore the role of *SLC7A11* in DCs, we screened differentially expressed genes in mregDC with and without *SLC7A11* expression and performed gene enrichment analysis. The results showed that upregulated genes were associated with the cell response to heat, whereas downregulated genes were associated with the pathway of DCs' differentiation (Figure S3D,E). Additionally, cell communication analysis revealed that mregDC shows a strong relative intensity in the CD80/CD86 pathway, indicating its potent co‐stimulatory capacity (Figure S4A,B). Compared with *SLC7A11* ^−^mregDC, *SLC7A11* ^+^mregDC showed a weaker co‐stimulatory score and lower *CD80/CD86* expression, suggesting reduced activation ability of CD4T cells (Figure S4C–E). Figure S4F–I show the corresponding ligand expression of CD4T and CD8T cell subsets. Related literature reported that in liver cancer patients who respond to immunotherapy, there is a cell triad: *CXCL13* ^+^CD4T cells and *PDCD1* ^+^ *CD8*T cells surround mregDCs, whereas there are no Treg cells in this area, suggesting that mregDC can play a role in positively regulating immunity in the liver cancer immune microenvironment [14].

### Targeting xCT in DCs combined with RFA to enhance anti‐tumor immunity

Given the significant upregulation of CD11c and xCT in the HCC microenvironment after RFA and the potential immunosuppressive effect of *SLC7A11*/xCT in DCs, we speculated that RFA combined with targeting *SLC7A11*/xCT in DC would be a potential combination therapy strategy. To verify this, we designed mice with knocking xCT in DC (CD11c^Cre^‐xCT^fl/fl^ mice) and control mice (xCT^fl/fl^ mice) (Figure S5).

**Figure 2:**
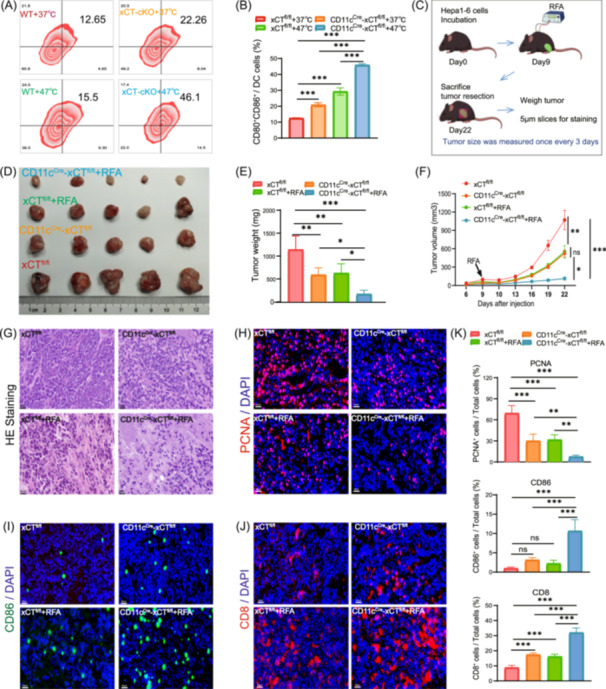
RFA combined with targeting *SLC7A11*/xCT in DCs is a potential anti‐tumor immune strategy. (A, B) Flow cytometry showing that heat treatment of BMDCs increases CD80^+^ and CD86^+^ DCs populations in xCT^fl/fl^ mice, with a stronger effect in CD11c^Cre^‐xCT^fl/fl^ mice. (C) Schematic of the animal model. (D) Representative images of tumors from different experimental groups showing smaller tumor sizes in CD11c^Cre^‐xCT^fl/fl^ mice compared to xCT^fl/fl^ mice after RFA treatment. (E) Tumor weight measurements further confirm the reduced tumor burden in CD11c^Cre^‐xCT^fl/fl^ mice post‐RFA compared to xCT^fl/fl^ mice. (F) Tumor growth curves indicate that RFA significantly slows tumor growth in CD11c^Cre^‐xCT^fl/fl^ mice compared to xCT^fl/fl^ mice. (G) Hematoxylin–eosin staining (HE) staining shows greater tumor cell destruction and reduced tumor cell density in CD11c^Cre^‐xCT^fl/fl^ mice treated with RFA compared to xCT^fl/fl^ mice. (H) Immunofluorescence staining for proliferating cell nuclear antigen (PCNA) indicates reduced tumor cell proliferation in CD11c^Cre^‐xCT^fl/fl^ mice, highlighting RFA's stronger anti‐tumor effect in this group. (I, J) CD86 and CD8 staining confirms enhanced co‐stimulatory and anti‐tumor immune responses in CD11c^Cre^‐xCT^fl/fl^ mice following RFA. (K) Fluorescence quantitative histograms (*p < 0.05; **p < 0.01; ***p < 0.001). DC, dendritic cell; RFA, radiofrequency ablation.

First, we extracted BMDCs from xCT^fl/fl^ mice and CD11c^Cre^‐xCT^fl/fl^ mice and compared the effect of heat treatment on CD80/CD86 expression in DCs. Experimental results showed that heat treatment significantly increased CD80/CD86 expression in xCT^fl/fl^ mice, and this effect was more significant in CD11c^Cre^‐xCT^fl/fl^ mice (Figure 2A,B). This suggests that xCT combined with heat treatment in targeted DCs has a synergistic effect on the co‐stimulation ability of DCs. In order to observe the overall effect of RFA in the tumor microenvironment of two groups of mice, we subcutaneously inoculated Hepa1‐6 cells into xCT^fl/fl^ mice and CD11c^Cre^‐xCT^fl/fl^ mice and treated them with RFA (Figure 2C). The stripped tumor volume, tumor growth curves, and tumor weight bar charts all showed that after RFA treatment, tumor regression in CD11c^Cre^‐xCT^fl/fl^ mice was more obvious than that in xCT^fl/fl^ mice (Figure 2D–F). Tumor tissue was sectioned and subjected to HE staining and PCNA staining. The results also showed the powerful anti‐tumor activity of RFA combined with xCT targeting DCs (Figure 2G–K). Similar to the results of heat‐treated BMDCs, CD86 expression in DCs was significantly increased in the liver cancer tumors of CD11c^Cre^‐xCT^fl/fl^ mice treated with RFA (Figure 2I,K). CD8^+^ T cells are the main effector cells responsible for killing tumor cells, so we performed CD8 immunofluorescence staining to compare the differences in CD8^+^ T cell activation. The results showed that RFA significantly activated CD8^+^ T cells in CD11c^Cre^‐xCT^fl/fl^ mice, with higher CD8 expression compared to the RFA‐only group, untreated CD11c^Cre^‐xCT^fl/fl^ mice, and xCT^fl/fl^ mice (Figure 2J,K).

## DISCUSSION

Through a comprehensive analysis, we elucidated the role of *SLC7A11* in DCs, notably in mregDC. However, the experimental design encompassed the global knockout of *SLC7A11*/xCT in all CD11c‐expressing DCs, not a targeted ablation specific to mregDC. Given that *SLC7A11* is predominantly expressed in mregDC and likely plays a more substantial role in the observed outcomes, the effects might nonetheless be confounded by off‐target impacts. This constitutes a limitation of our study; hence, the experimental findings should be interpreted as reflecting the broader role of *SLC7A11* in DC functionality. Furthermore, although evidence suggests that xCT in DCs can suppress CD80/CD86 expression, it is important to note that xCT, as a transporter protein, does not exert direct control over downstream proteins. The influence on co‐stimulatory molecule expression is likely mediated through diverse pathways, and this study's limited exploration of the underlying mechanisms is a recognized limitation. Ultimately, although targeting *SLC7A11* augments the co‐stimulatory potential of DCs, the cytotoxic effects of RFA's high temperatures might counteract this by impairing DC functionality, consequently diminishing their immunomodulatory capacity. Therefore, the optimal time window for combining these two treatment methods requires further investigation.

Therefore, future research should aim to develop more targeted approaches to further explore the mechanisms by which xCT inhibition leads to the upregulation of co‐stimulatory molecules. Additionally, studies should investigate the optimal time window for combining these two treatment methods and explore the potential feasibility of incorporating immune checkpoint inhibitors.

## CONCLUSION

In conclusion, our study underscores the therapeutic potential of targeting *SLC7A11* in tandem with RFA to bolster antitumor immunity in hepatocellular carcinoma. We posit that this approach merits further exploration and may pave the way for more efficacious treatment strategies for patients with HCC.

## AUTHOR CONTRIBUTIONS

**Yuzhao Jin**: Writing—original draft; project administration; visualization. **Songhua Cai**: Validation; investigation; data curation; writing—review and editing. **Yang Zhou**: Writing—review and editing; validation. **Dandan Guo**: Data curation; validation; investigation; writing—review and editing. **Yuzhen Zeng**: Data curation; validation; investigation; writing—review and editing. **Wangting Xu**: Investigation; validation; data curation; writing—review and editing. **Yiting Sun**: Validation; investigation; writing—review and editing. **Yueli Shi**: Writing—review and editing; software. **Zhiyong Xu**: Writing—review and editing; software. **Zaoqu Liu**: Writing—review and editing; software. **Peng Luo**: Data curation; project administration; formal analysis; supervision; writing—review and editing. **Zhao Huang**: Validation; formal analysis; supervision; writing—review and editing. **Bufu Tang**: Funding acquisition; writing—review and editing; validation; data curation; supervision; resources.

## CONFLICT OF INTEREST STATEMENT

The authors declare no conflicts of interest.

## ETHICS STATEMENT

All animal experiments conducted in this study were approved by the Ethics Committee of the Cancer Hospital Chinese Academy of Medical Sciences (No. NCC2024A560).

## Supporting information

[Correction added on 29 November 2024, after first online publication: The supporting information file has been updated for improved clarity and thoroughness.]

**Figure S1:** Impact of RFA on xCT upregulation in DCs and the prognostic significance of SLC7A11 in liver cancer.
**Figure S2:** Supplementary single‐cell analysis part 1.
**Figure S3:** Supplementary single‐cell analysis part 2.
**Figure S4:** Cell communication analysis and ligand/receptor display.
**Figure S5:** Specific SLC7A11 knockout in mouse DCs

## Data Availability

The data that support the findings of this study are available in the supplementary material of this article. The sources of data used in this study are summarized in Materials and Methods, and all data are publicly available for download. The data and scripts used are saved in GitHub https://github.com/yuzhaojin/xCT. Supplementary materials (methods, figures, scripts, graphical abstract, slides, videos, Chinese translated version, and update materials) may be found in the online DOI or iMeta Science http://www.imeta.science/.
