# Supplementary material for: Targeting SLC7A11/xCT improves radiofrequency ablation efficacy of HCC by dendritic cells mediated anti‐tumor immune response
Source: Imeta. 2024 Nov 20;3(6):e248. doi: 10.1002/imt2.248 (PMC11683471; doi:10.1002/imt2.248)
Supplement: Supplementary file 1 — Figure S1: Impact of RFA on xCT upregulation in DCs and the prognostic significance of SLC7A11 in liver cancer. Figure S2: Supplementary single‐cell analysis part 1. Figure S3: Supplementary single‐cell analysis part 2. Figure S4: Cell communication analysis and ligand/receptor display. Figure S5: Specific SLC7A11 knockout in mouse DCs [file IMT2-3-e248-s001.doc]

**Supporting information to**

Targeting *SLC7A11*/xCT improves radiofrequency ablation efficacy of HCC by dendritic cells mediated anti-tumor immune response

**Running title:** Targeting *SLC7A11* Enhances Immune Response in HCC Ablation

Yuzhao Jin^1#^, Songhua Cai^2#^, Yang Zhou^3#^, Dandan Guo^4^, Yuzhen Zeng^5^, Wangting Xu^6^, Yiting Sun^7^, Yueli Shi^8^, Zhiyong Xu^8^, Zaoqu Liu^9^, Peng Luo^10*^, Zhao Huang^11,12*^,Bufu Tang^4,5*^

^1^Postgraduate Training Base, Wenzhou Medical University, Wenzhou, China

^2^Department of Thoracic Surgery, National Cancer Center/National Clinical Research Center for Cancer/Cancer Hospital & Shenzhen Hospital, Chinese Academy of Medical Sciences and Peking Union Medical College, Shenzhen, China

^3^Department of Gynecologic Oncology, Zhongshan Hospital, Fudan University, Shanghai, China

^4^Department of Oncology, First Affiliated Hospital, Dalian Medical University, Dalian, China

^5^Department of Radiation Oncology, Zhongshan Hospital Affiliated to Fudan University, Shanghai, China

^6^Department of Respiratory Medicine, Sir Run Run Shaw Hospital, Zhejiang University, Hangzhou, China

^7^Department of Clinical Medicine, China Medical University, Shenyang, China

^8^Department of Respiratory and Critical Medicine, Center for Oncology Medicine, The Fourth Affiliated Hospital of School of Medicine, Zhejiang University, Yiwu City, China

^9^Institute of Basic Medical Sciences, Chinese Academy of Medical Sciences and Peking Union Medical College, Beijing, China

^10^Department of Oncology, Zhujiang Hospital, Southern Medical University, Guangzhou, Guangdong, China

^11^Hepatic Surgery Center, Tongji Hospital, Tongji Medical College, Huazhong University of Science and Technology, Wuhan, China

^12^Hubei Key Laboratory of Hepato-Pancreatic-Biliary Diseases, Tongji Hospital, Tongji Medical College, Huazhong University of Science and Technology, Wuhan, China

^#^These authors contributed equally: Yuzhao Jin, Songhua Cai, Yang Zhou

*Correspondence: tangbufu@zju.edu.cn (Bufu Tang); huangzhao@tjh.tjmu.edu.cn (Zhao Huang); luopeng@smu.edu.cn (Peng Luo)

**
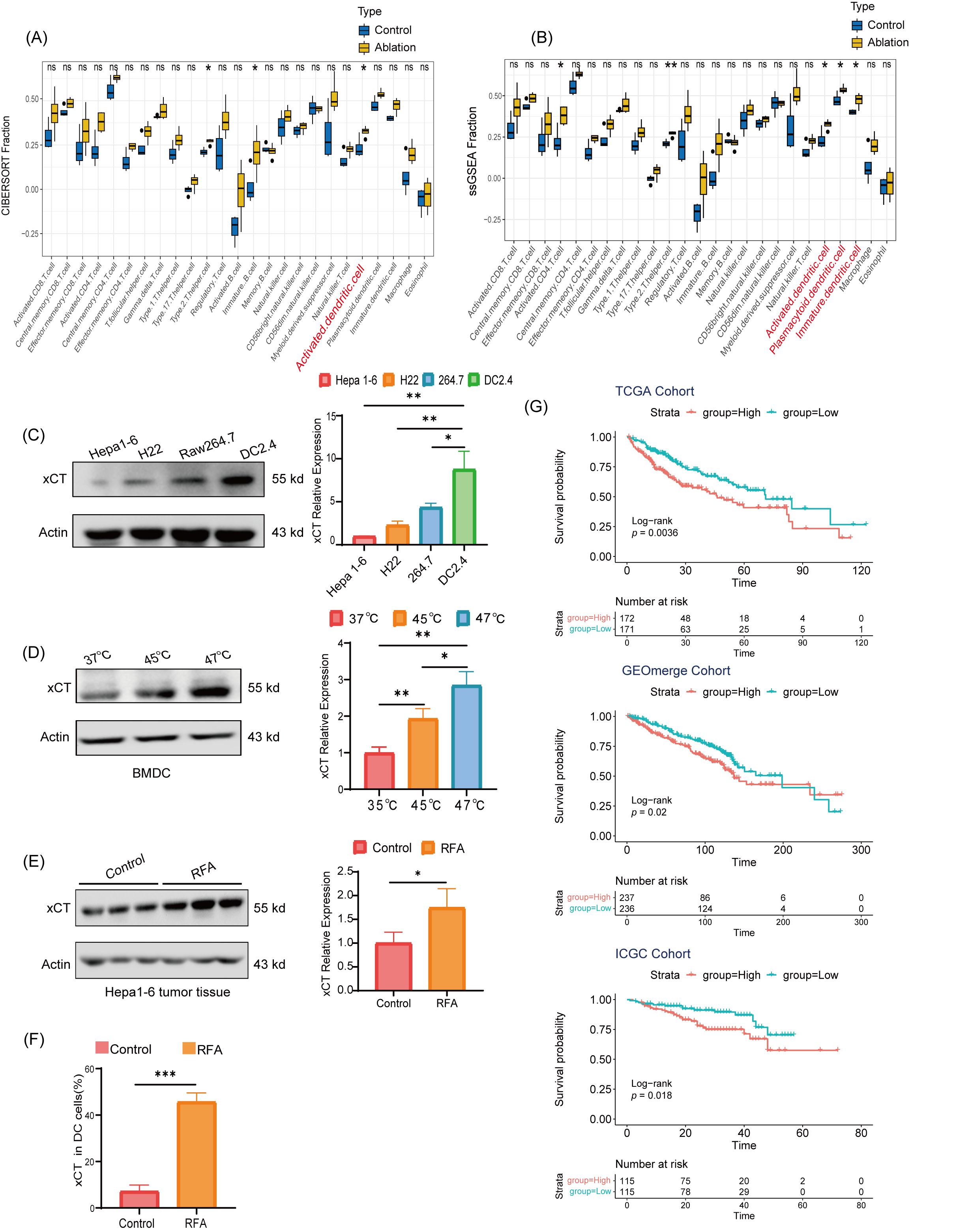
**

**Figure S1 Impact of RFA on xCT upregulation in DCs and the prognostic significance of *SLC7A11* in liver cancer.** (A, B) Box plots indicate the comparative proportions of immune cells in tumor tissues, as determined by Single-sample gene set enrichment analysis (ssGSEA) and Cell-type Identification By Estimating Relative Subsets Of RNA Transcripts (CIBERSORT). (C) Western blot analysis reveals higher xCT protein levels in the Dendritic Cells (DCs) compared to TAMs and liver cancer cell lines. (D) Western blot analysis indicates increased xCT expression in Marrow-Derived Dendritic Cells (BMDCs) in response to elevated treatment temperatures. (E) Western blot confirms upregulation of xCT post-Radiofrequency ablation (RFA) in Hepa1-6 subcutaneous tumor models. (F) Bar graph representing the ratio of xCT-expressing DCs in subcutaneous tumors of mice with and without RFA treatment. (G) Analysis of The Cancer Genome Atlas (TCGA), Gene Expression Omnibus data base (GEO) merge and International Cancer Genome Consortium (ICGC) cohorts establishes SLC7A11 as a poor prognosis marker in Hepatocellular carcinoma (HCC). (Significance: **p* < 0.05, ***p* < 0.01, ****p* < 0.001).

**
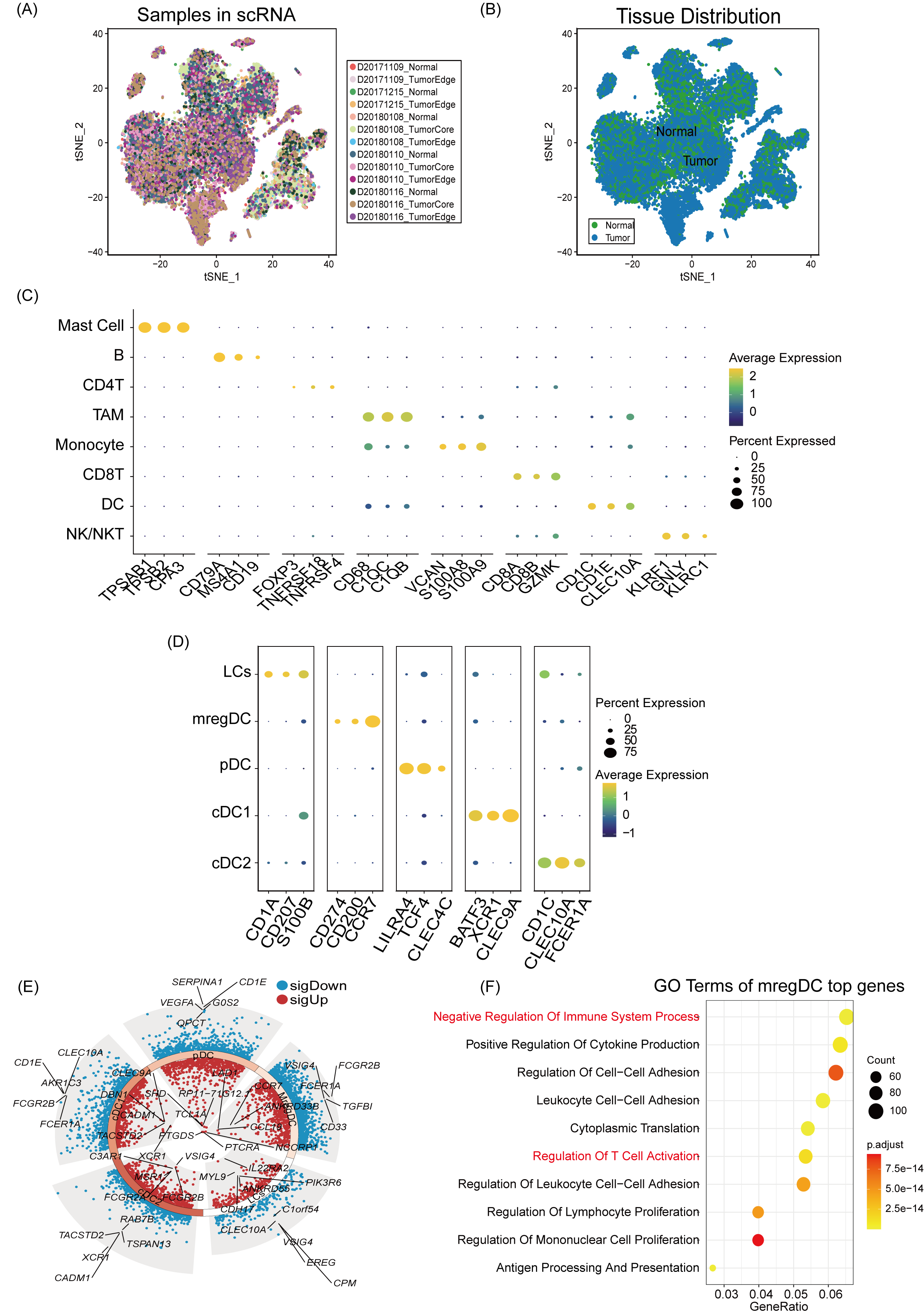
**

**Figure S2 Supplementary single-cell analysis part 1.** (A) tSNE map depicting Single-cell RNA (scRNA) sample origins. (B) tSNE map illustrating the tissue distribution of scRNA samples. (C) Dot plot displaying the expression levels of marker genes across various cell types. (D) tSNE maps displaying marker genes for various immune cells. (E) Volcano plot of the top 5 differentially expressed genes in each DCs group. (F) Dot plot presenting Gene Ontology (GO) analysis results for top mregDC genes.


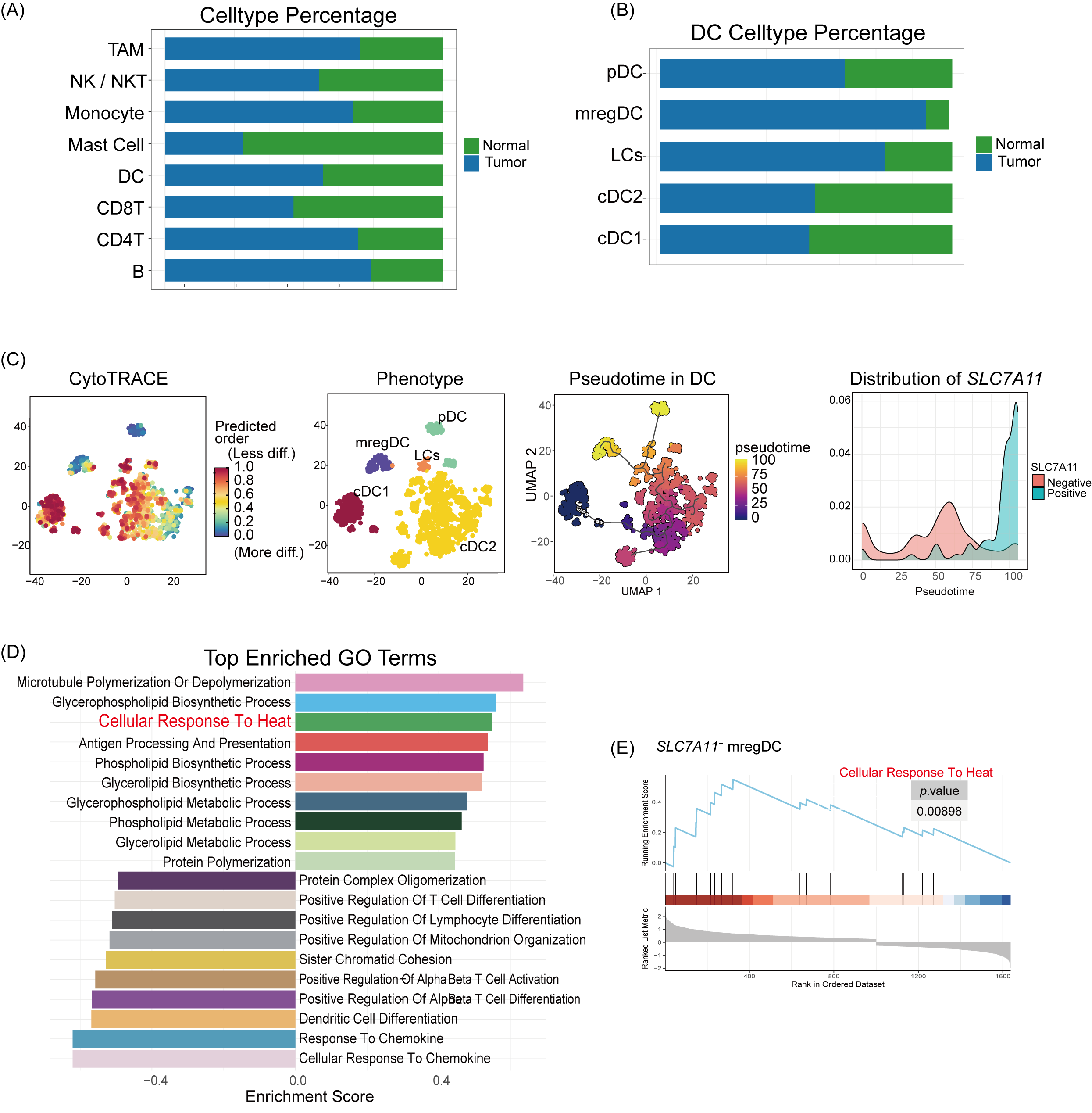


**Figure S3 Supplementary single-cell analysis part 2.** (A) Stacked bar chart showing cell-type proportions in normal vs. tumor tissues. (B) Stacked bar chart comparing the proportions of DCs subtypes in normal vs. tumor tissues. (C) Pseudotime analysis revealing mregDC at the terminal stage of DCs differentiation, where *SLC7A11* expression is also elevated. (D) Gene Set Enrichment Analysis (GSEA) shows key pathways differentially enriched in mregDC with or without *SLC7A11* expression. (E) GSEA diagram indicating significant enrichment of differentially expressed genes from *SLC7A11^+ / -^*mregDC in the Cellular Response to Heat pathway.

**
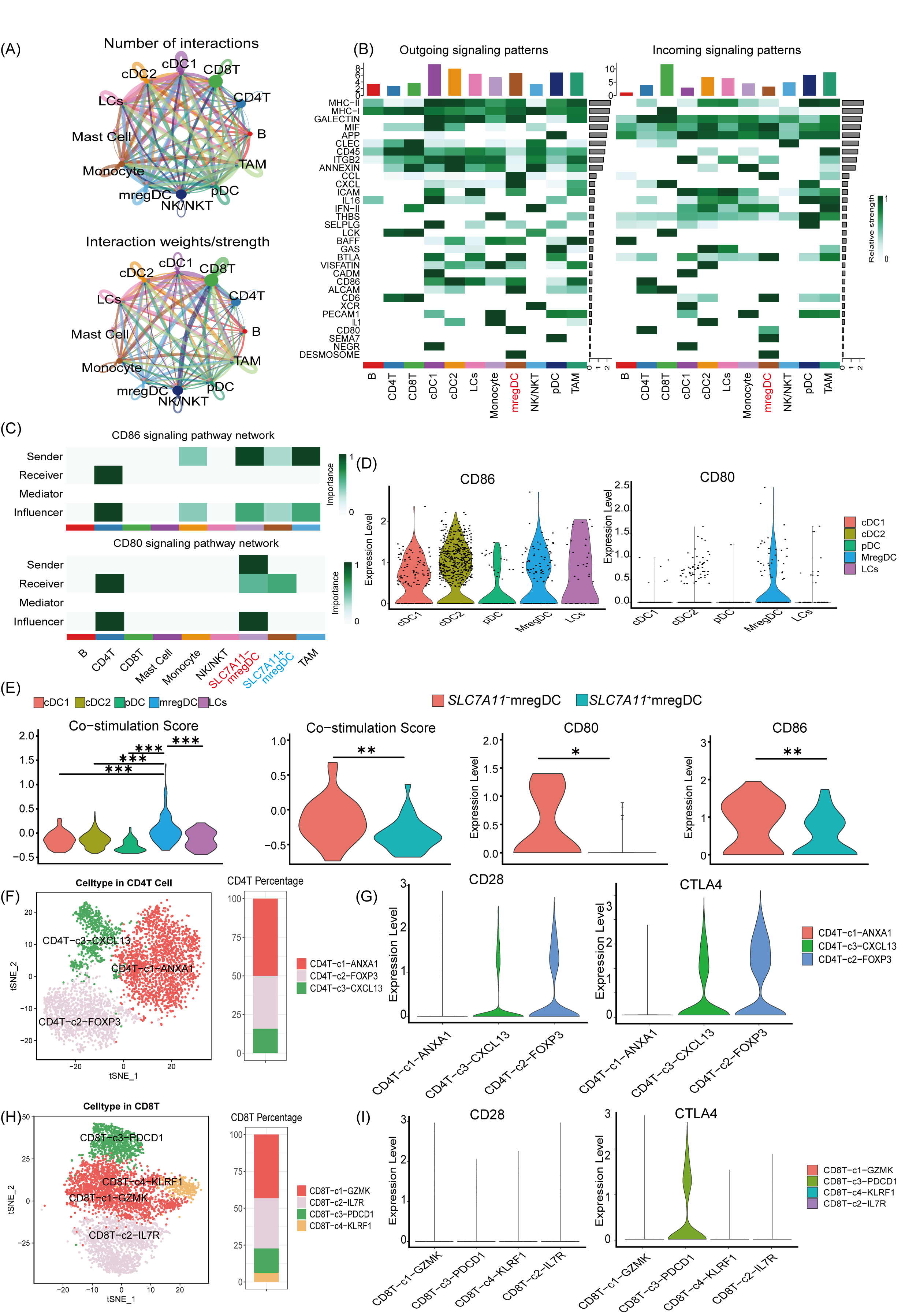
**

**Figure S4 Cell communication analysis and ligand/receptor display** (A) Cellular interaction circular network diagram. (B) Heatmap showing signaling patterns between different cell types. (C) Heat map showing the communication between *SLC7A11*^+/-^mregDC and other cells. (D) Violin plots showing the expression of *CD80* and *CD86* in different types of DCs (E) Violin plots reveal mregDC has the highest co-stimulation score, with *SLC7A11*^-^ mregDC showing higher co-stimulation and expression of *CD80* and *CD86* compared to *SLC7A11*^+^ mregDC. (F) tSNE diagram showing the clustering of CD4 T cells and the top differentially expressed gene in the clusters. (G) Violin plots showing the expression of *CD28*, *CTLA4* in CD4 T cells. (H) tSNE diagram showing the clustering of CD4 T cells and the top differentially expressed gene in the clusters. (I) Violin plots showing the expression of *CD28*, *CTLA4* in CD8 T cells. (Significance: **p* < 0.05, ***p* < 0.01, ****p* < 0.001).

**
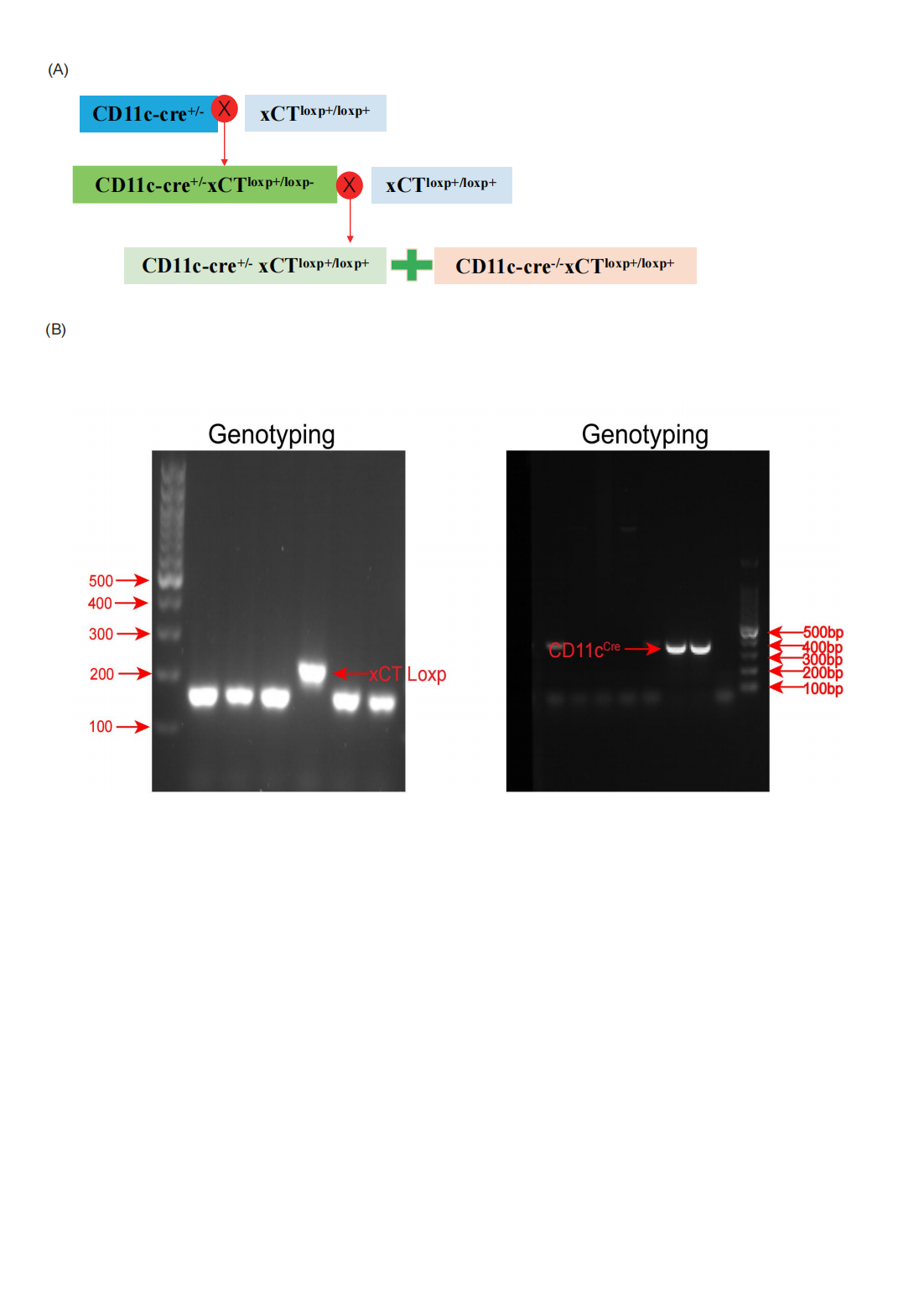
**

**Figure S5 Specific *SLC7A11* knockout in mouse DCs** (A) The hybridization strategy diagram of CD11c^Cre^-xCT^fl/fl^ mice. (B) Polymerase Chain Reaction (PCR) assay showing the genotyping result of the CD11c^Cre^-xCT^fl/fl^ mice and xCT^fl/fl^ mice.

**Materials and Methods**

**Data acquisition**

Bulk RNA raw data were sourced from the PRJNA815907 project, encompassing matched liver cancer tissues pre- and post-ablation. We performed quality control and trimming using trim_galore, aligned using hisat2, obtained counts using samtool and featureCounts, and obtained the gene expression matrix after standardization. Survival data collection encompassed publicly available datasets, including The Cancer Genome Atlas (TCGA), the International Cancer Genome Consortium (ICGC) and Gene Expression Omnibus data base (GEO) merge cohorts. GEO data utilized for Kaplan-Meier Survival (KM analysis) were derived from GSE10143, GSE15654, and GSE76427, with batch effects mitigated using removeBatchEffect. The Single-cell RNA (scRNA) data from GSE140228 collects CD45+ immune cells from liver cancer patient tissues

**CIBERSORT and ssGSEA**

Gene expression data analysis was conducted using Cell-type Identification By Estimating Relative Subsets Of RNA Transcripts (CIBERSORT). The input data consisted of normalized gene expression values obtained from the PRJNA815907 project. We used the LM22 gene signature, which defines 22 human immune cell phenotypes, as the reference for cell type deconvolution. The output provided the relative proportions of each immune cell type within the samples.

Single-sample Gene Set Enrichment Analysis (ssGSEA) was implemented to evaluate the enrichment of predefined gene sets per sample. We used the immunecell-related gene sets. Normalized gene expression data served as the input for ssGSEA. Enrichment scores for each gene set were calculated, reflecting the degree to which the genes in a particular set are coordinately up- or down-regulated within each sample. These scores are then used to compare the proportions of different cells in the samples.

**scRNA data process**

Analysis of the GSE140228 cohort was facilitated by the Seurat R package, employing preprocessing steps to ensure high data quality. Initially, cells were filtered based on the following criteria: each cell must express a minimum of 400 unique features, total RNA counts must range between 1,000 and 20,000, and the proportion of mitochondrial gene content must be less than 10%. Seurat objects were then generated for tumor and normal tissue samples. Data normalization was conducted using the NormalizeData function, followed by scaling using the ScaleData function. The 2,000 most highly variable genes were selected for downstream analysis, and principal component analysis (PCA) was performed. Based on elbow plot inspection, the optimal number of principal components (PCs) was determined to be 30.

To account for batch effects, the Harmony algorithm was applied to correct for technical variations across samples. Clustering of cells was performed at a resolution of 1.0, and cell populations were manually annotated using established marker genes. Visualization of the clusters was achieved through tSNE plots.

For further analysis, DCs populations as well as *CD4*^+^ and *CD8*^+^ T cells were isolated. These populations underwent a secondary round of normalization and processing using the same pipeline. Clustering was then conducted at a finer resolution of 0.3, followed by manual annotation to assign specific cell population identities based on known marker expression.

**CytoTRACE and Pseudotime analysis**

Cellular Trajectory Reconstruction Analysis using gene Counts and Expression (CytoTRACE) was employed to assess the differentiation degree of various DCs, with lower scores indicating higher differentiation levels. Monocle 3 was used to infer pseudo-time trajectories, with cDC1, the least differentiated cell, as the developmental starting point for trajectory inference, and the DDRTree algorithm was used to construct trajectories. Cells were arranged along the pseudo-time axis to represent the changes in *SLC7A11* during the dynamic process.

**Enrichment analysis and module scoring**

Gene set enrichment analysis (GSEA) was conducted utilizing the clusterProfiler package. Gene sets from the Molecular Signatures Database (MSigDB) were used to assess the enrichment of biological pathways. Differentially expressed genes were ranked by their expression levels. Enrichment scores were calculated to identify significantly enriched pathways.

Co-stimulatory signature activity in DCs was assessed using the AddModuleScore function in the Seurat R package. Co-stimulatory signature gene sets include *CD80、CD86、CD40、TNFSF4、PVR.*

**Cell communication analysis**

Cell-to-cell communication analysis was conducted utilizing CellChat and CellPhoneDB. Infer receptor-ligand interactions from scRNA-seq data based on known interaction databases. Extract expression data for receptor-ligand pairs and calculate interaction scores. Perform network visualization to illustrate communication patterns between cell types or clusters.

**Generation of BMDCs and heat treatment**

Bone marrow cells were extracted from the femur and tibia of mice and depleted of erythrocytes using Ammonium-Chloride-Potassium lysis buffer (Beyotime). The remaining cells were seeded at a concentration of 1 × 10^6 cells/ml in RPMI-1640 medium (Gibco) supplemented with 10% fetal bovine serum (FBS), 50 U/ml penicillin , 50 U/ml streptomycin , 10 ng/ml mrGM-CSF (Peprotech) and 10 ng/ml IL-4 (Peprotech). Cells were incubated at 37°C in a humidified atmosphere with 5% CO2 for 7 days, with media and cytokines replenished every two days. On day 7, non-adherent cells were collected and purified using anti-mouse CD11c magnetic beads (Miltenyi Biotec) with an AutoMacs device (Miltenyi Biotec). The purified immature dendritic cells were then treated with 100 ng/ml Lipopolysaccharide (LPS) (Sigma) for 24 hours. Preheat the water bath in advance. Wrap the Marrow-Derived Dendritic Cells (BMDCs) culture bottle with aluminum foil and immerse it completely in water for 1 hour. After the cells cool to normal temperature, use them for other experiments.

**Cell Culture**

Hepa1-6, H22, Raw264.7 and DC2.4 cell lines were cultured under standard conditions. Hepa1-6 cells were grown in DMEM, H22 in RPMI-1640, Raw264.7 in DMEM and DC2.4 in RPMI-1640, all supplemented with 10% FBS and 1% penicillin-streptomycin. Cells were incubated at 37°C with 5% CO2. Subculturing was performed at 70-80% confluency by washing with Phosphate buffered saline (PBS) and detaching with 0.05% trypsin-Ethylenediaminetetraacetic acid (EDTA), followed by neutralization with complete medium, centrifugation, resuspension and reseeding at the desired density.

**Western Blot (WB)**

Prepare Sodium dodecyl sulfate polyacrylamide gel electrophoresis (SDS-PAGE) gel and corresponding cell or tumor tissue lysate. After electrophoresis, transfer the imprint to a Polyvinylidene fluoride (PVDF) membrane, then block with 5% skim milk at room temperature for one hour. Select an appropriate ratio and incubate with primary antibody overnight in a 4°C shaker. Wash the membrane the next day and select an appropriate secondary antibody for incubation. Wash the membrane again and develop with chemiluminescence and take pictures.

**Construction of mouse subcutaneous tumor model and RFA treatment**

1×10^6 cells/100 µL Hepa1-6 cells were subcutaneously injected into the inner side of the left lower limb of 8-week-old male C57BL/6 mice. When the tumor reached about 100 mm^3^, an 18G bipolar ablation needle was placed into the tumor tissue, and the Radiofrequency ablation (RFA) tail needle was connected to the RF transmitter. The RFA temperature was set to 60°C, and RFA was repeated for 2 minutes. The mouse tumor was removed the next day, part of which was used to extract tissue protein for WB, and part of which was used for immunofluorescence detection of the co-expression of xCT and *CD11c*.

1×10^6 cells/100 µL Hepa1-6 cells were subcutaneously injected into the inner side of the left lower limb of 8-week-old male CD11c^Cre^-xCT^fl/fl^ (Itgax^Cre^-xCT^fl/f^) C57BL/6 mouse models and control xCT^fl/fl^ C57BL/6 mice. On the ninth day, the same ablation treatment was performed. The tumor volume was measured the next day, and the fluid volume was measured every three days thereafter. The mice were killed on the 22^nd^ day. After the tumor was removed, it was photographed and weighed, fixed in formalin for 24 hours, dehydrated with gradient ethanol, embedded in paraffin and sliced for subsequent experiments.

Tumor volume = (½ × long diameter) × short diameter ^2^. The diameter of the tumor was measured with a caliper. 6-8 week old C57BL/6 male mice were purchased from Shanghai Experimental Animal Center (Shanghai, China). CD11c^Cre^-xCT^fl/fl^ C57BL/6 mouse models and control xCT^fl/fl^ C57BL/6 mice were purchased from by Cyagen Biosciences Inc. All mice were housed according to Specific Pathogen Free (SPF) standards, maintained at 24°C ± 2°C, 40%-70% relative humidity, 12 h light and 12 h dark cycle and prepared for animal experiments.

**Flow Cytometry (FC)**

The cells were collected and resuspended in PBS containing 2% FBS. After centrifugation, the supernatant was removed and the appropriate antibody dilution was added for incubation at 4°C in the dark for 30 minutes. After the incubation, the cells were washed twice with PBS and resuspended in 500 µL PBS for detection. Data were collected using a flow cytometer and analyzed using FlowJo software.

**Immunofluorescence (IF) Staining**

Sections 4–6 µm thick were cut from tumor paraffin blocks and placed on glass slides. Sections were dewaxed in xylene, rehydrated through a decreasing series of ethanol and subjected to antigen retrieval using citrate buffer (pH 6.0) in a microwave oven. After cooling to room temperature, sections were blocked with 5% bovine serum albumin (BSA) for 1 hour. Primary antibodies were applied and incubated overnight at 4°C. After washing with PBS, sections were incubated with fluorescently labeled secondary antibodies for 1 hour at room temperature in the dark. Nuclei were counterstained with 4’,6-Diamidino-2’-phenylindole (DAPI) and mounted with antifade mounting medium. Fluorescence images were captured using a fluorescence microscope.

**Hematoxylin and Eosin (HE) Staining**

The sections were deparaffinized in xylene and rehydrated through a descending ethanol series. Slides were then stained with hematoxylin for 5 minutes, rinsed in running tap water for 5 minutes, differentiated in 1% acid alcohol for a few seconds and blued in 0.2% ammonia water or saturated lithium carbonate solution. After rinsing in tap water, sections were counterstained with eosin for 2 minutes. The stained sections were dehydrated through an ascending ethanol series, cleared in xylene and mounted with a coverslip using a synthetic resin mounting medium. The slides were examined and imaged using a light microscope.

**Statistical analysis**

Data were expressed as mean ± SEM. KM survival curves were generated to compare survival distributions between different groups, and the log-rank test was employed to determine statistical significance. Univariate Cox proportional hazards models were used to estimate the hazard ratios and 95% confidence intervals for *SLC7A11* expression. Kruskal.test and Dunn's Test were used to examine the co-stimulation scores across multiple groups in the single-cell gene matrix, with the R packages ggpubr and FSA, respectively. The Wilcox test was used to examine differential gene expression and scores between two groups, using the R package ggpubr. ANOVA and Tukey's test were used to assess differences in data across multiple groups in the experiment. The two-tailed Student's t-test was used to examine data differences between two groups in the experiment. The R version used was 4.2, and the GraphPad Prism version was 9.5. *p* values < 0.05 were considered significant (*p* < 0.05*; *p* < 0.01**, *p* < 0.001***).

**Reagent Description**

In the WB experiment, we selected β-Actin (mouse, Abcam, ab8226, 1:4000) and xCT (rabbit, Abcam, ab307601, 1:1000).

In the IF experiment, we used CD11c (mouse, Abcam, ab255937, 1:100), xCT (rabbit, Abcam, ab307601, 1:500), PCNA (rabbit, Cell Signaling Technology, #13110, 1:400), CD8 (rabbit, Abcam, ab217344, 1:100) and CD86 (rabbit, Abcam, ab239075, 1:100).

In the FC experiment, we used APC-labeled CD80 (clone 16-10A1, Biolegend, 104714) and FITC-labeled CD86 (clone GL-1, Biolegend, 105006). All antibodies were used at the recommended dilution ratios to ensure the accuracy and reliability of the experimental results.
